# Extramedullary malignant melanotic schwannoma of the spine: Case report and an up to date systematic review of the literature

**DOI:** 10.1016/j.amsu.2020.10.003

**Published:** 2020-10-07

**Authors:** Georgios Solomou, Adikarige Haritha Dulanka Silva, Adrianna Wong, Ute Pohl, Nikolaos Tzerakis

**Affiliations:** a School of Medicine, Keele University, Staffordshire, UK Hospital Campus, Newcastle Road, Stoke-on-Trent ST4 6QG, UK; b Paediatric Neurosurgery Fellow, Department of Neurosurgery, Great Ormond Street Hospital for Children NHS Foundation Trust, Great Ormond Street, London, WC1N 3JH, UK; c School of Medicine, Keele University, UK Hospital Campus, Newcastle Road, Stoke-on-Trent, Staffordshire, UKST4 6QG, UK; d Consultant Neuropathologist, University Hospitals Birmingham NHS Foundation TrustQueen Elizabeth Hospital Birmingham, Mindelsohn Way, Edgbaston Birmingham, B15 2GW, UK; e Consultant Neurosurgeon, University Hospital of North Midlands, Stoke on Trent, ST4 6QG, UK

**Keywords:** Extramedullary, Melanotic, Malignant, Metastatic, Schwannoma

## Abstract

**Background:**

Melanotic schwannoma is a rare variant of schwannoma. Extramedullary melanotic schwannoma originates in the vicinity of nerve roots mimicking other intervertebral disc disorders. Therefore, T1 and T2-weighted MRI sequences become an essential tool for diagnosis. Aside from case reports, no large studies exist to provide consensus on the signal intensities in T1 and T2-weighted MR imaging. Moreover, no clear evidence is available to delineate prognosis. Here, a case report is presented together with a subsequent systematic review of the literature regarding this rare entity.

**Case description:**

A 45-year old female presented with a one-year history of insidious onset of neck pain and paraesthesia. Magnetic resonance imaging confirmed an extramedullary lesion along the C6 nerve root with T1-weighted hyperintensity and T2-weighted hypointensity. Despite two surgical decompressions and adjuvant immunotherapy, the patient unfortunately passed away due to metastatic progression.

**Discussion:**

According to the systematic review conducted, in over half of the cases of extramedullary melanotic schwannoma, there is local reoccurrence and/or distal metastasis. Moreover, in 64.7% and 70.6% of the cases, the T1-weighted image of the lesion appears hyperintense and hypointense on a T2-weighted image, respectively. It is an aggressive variant of schwannoma, one of the most commonly observed extramedullary tumours presenting to neurosurgical practice.

**Conclusion:**

Our results highlight that specific T1 and T2-weighted imaging findings can provide valuable information, enabling early suspicion, influencing the surgical aims and strategy and the timely commencement of relevant immunotherapy. Considering the poor prognosis, early adjuvant therapy with other modalities should be considered.

## Highlights

• Up to date review collating evidence regarding local recurrence/metastasis and MR imaging findings unique for the melanotic schwannoma.
• The lesion appears hyperintense and hypointense on a T1-weighted and T2-weighted magnetic resonance imaging, respectively.
• In over half of the cases, there is local recurrence and distal metastasis thus a radical excision is recommended early on.
• Neurosurgeons should embrace discussion with oncologist for early adjuvant therapy.

## 1 Introduction

Schwannomas are solitary, encapsulated and slow-growing neoplasms composed of Schwann cells constituting the nerve sheath [1]. Melanotic schwannoma (MeS) or melanocytic schwannoma, initially described by Millar in 1932 [2], is a rare variant composed of neoplastic Schwann cells that produce melanin and account for less than 1% of primary peripheral nerve sheath tumours [1]. It is hypothesized that Schwann cells are capable of synthesizing melanin, owing to a common progenitor of both Schwann cells and melanocytes being migrating neural crest cells [3]. Macroscopically, they appear black, brown or dark blue in colour, whereas under light microscopy, there is evidence of heavy melanin deposition, spindle morphology, nuclear pleomorphism and low mitotic rate with or without psammoma bodies [4,5]. To date, approximately 200 cases of MeS have been described in the literature, predominantly as case reports, but also some small case series. Posterior nerve roots represent the most frequently involved site (30.5%) [6], but MeS affecting the spinal cord, sympathetic chain, cranial nerve roots, peripheral nerves and the gastrointestinal tract [[7], [8], [9], [10], [11], [12]] have also been documented. It is generally accepted that MeS can be divided into psammomatous and non-psammomatous [[13], [14], [15]] subtypes. In 1990, Carney described the psammomatous MeS as a distinct clinicopathologic entity and proposed it be considered as part of the Carney complex in conjunction with the presence of cutaneous lesions, endocrine tumours and cardiac myxoma [13]. A Mendelian dominant hereditary pattern was then defined as being responsible for the clinical phenotype [16]. Extramedullary MeS presents in similar fashion to other extramedullary spinal tumours. Originating in the vicinity of the spinal nerve roots, it often leads to compression within and narrowing of the intervertebral foramen, leading to the manifestations of radicular pain, back pain, dysesthesias and progressive sensory and motor deficits. The non-specific combination of radicular pain and back pain can often lead to the clinical misdiagnosis of an intervertebral disc disorder. As the tumour grows, it leads to the progression of motor and sensory signs with Magnetic Resonance Imaging (MRI) guided assessment, an important diagnostic tool [17].

Until 1998, the MRI characteristics of MeS of the spine had not been described. Bendszus et al. originally described the lesion to appear hyperintense in T1 and hypointense in T2 [18]. There have been no extensive reviews focusing on the imaging of MeS, although it has been globally accepted that it has a characteristic hyperintensity on T1-weighted sequence and hypointensity on T2-weighted sequence [19]. Clinically, several studies have commented on the malignant and metastatic potential of MeS, however, with heterogeneous outcomes [12,51,57]. We describe and discuss a case of rare fulminant MeS with distal and local metastases, and provide a systematic review of both imaging as well as prognostic outcomes regarding MeS, including highlighting its metastatic potential.

## 2 Case report

### 2.1 History

A 45-year-old female patient of white ethnic background was referred to the neurosurgical service by the spinal physiotherapy department. She presented with a one-year history of insidious neck pain radiating to the shoulder girdle, left arm and thumb, with associated paraesthesia in the same distribution. The pain was worse on resting and relieved by non-steroidal anti-inflammatory drugs (NSAIDs). An initial diagnosis of C6 radiculopathy was made and, given the failure of conservative management, she was offered a contrast-enhanced MRI. The only relevant past medical history was that of hiatus hernia and thyroid adenoma for which she had a partial thyroidectomy 10 years previously, with levothyroxine as the only regular medication. No family history of malignancy was found.

### 2.2 Radiological findings

**Fig. 1:**
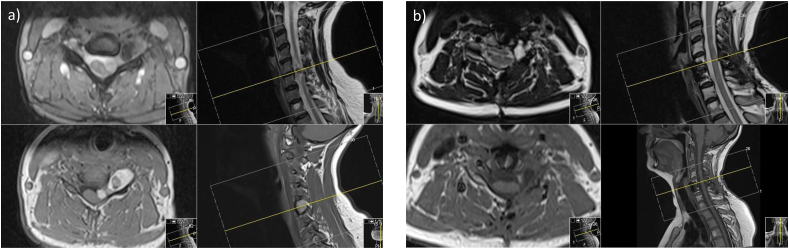
A) Pre-operative magnetic resonance imaging (MRI) in T2 (superior plates) and T1 (inferior plates) weighted imaging sequences. Axial (left) and Sagittal (right) sequences presented. The left-sided dumbbell shaped lesion is visualised extending through the intervertebral foramen with both intra-dural intra-canalicular and extra-dural extra-canalicular elements. The T1 weighted sequence demonstrates the characteristic ‘target’ sign with central hypo-intensity with circumferential peripheral enhancement. b) Post-operative MRI in similar configuration demonstrating near total excision of the lesion. A small focus of contrast-enhancement intra-dural and intra-canalicular is visualised representing possible residual remnant.

Cervical spine imaging revealed a dumbbell-shaped lesion along the C6 nerve root, with both intradural and extradural components. The central portion of the extradural component, in particular, demonstrated T2 hypointensity on most sequences, while the peripheral portions enhanced homogeneously (‘target’ sign, consistent with a peripheral nerve sheath tumour [usually suggestive of neurofibroma, but also seen in schwannomas and malignant peripheral nerve sheath tumours]) [20]. The overall appearances were consistent with schwannoma (Fig. 1).

### 2.3 Surgical approach/management

Following discussion at the neuro-oncological multi-disciplinary team (MDT) meeting, a recommendation for surgical excision was made. A C5/6 left hemilaminectomy was performed under intra-operative neurophysiological monitoring (IONM). Post-operatively, the patient showed improvement of symptoms in the left arm.

### 2.4 Histopathological findings

Histology revealed a heavily pigmented spindle cell neoplasm, arranged in solid sheets and fascicles, with focal epitheloid differentiation and several well-defined areas of necrosis. The individual tumour cells displayed nuclear pleomorphism with prominent nucleolation and well-defined basophilic cytoplasm with finely granular brown pigment consistent with neuromelanin. Mitoses amounted to 3/single high power field (×40 objective). Psammoma bodies were absent. Immunohistochemistry showed a nuclear labelling index for the proliferation marker ki-67 of up to 25%, and strong staining for S100, MelanA and HMB45, while pancytokeratin CK AE1/3 was negative. Histochemistry for reticulin demonstrated predominantly pericellular collagen staining in the tumour, while focal loss of pericellular reticulin deposition was noted in some areas. A diagnosis of ‘malignant melanotic spindle cell neoplasm, consistent with malignant melanotic schwannoma’, based on the anaplastic histological features in this biopsy, was confirmed.

The latest edition of the WHO classification of tumours of the central nervous systems (2016) has not yet assigned a grade for this variant, but its aggressive behaviour reported in the literature suggests a grade of at least WHO III. Genetic analysis was negative for BRAF V600 mutation, and PD-L1 expression was absent. Further genetic studies were not undertaken. Although the histology was most in keeping with a malignant melanotic schwannoma, at MDT, a dermatological review was recommended to rule out the possibility of metastatic malignant melanoma.

### 2.5 Further management

In light of histology and MDT discussion, the patient was referred to dermatology for an urgent review and staging computerised tomography (CT) scan of the chest, abdomen and pelvis.

**Fig. 2:**
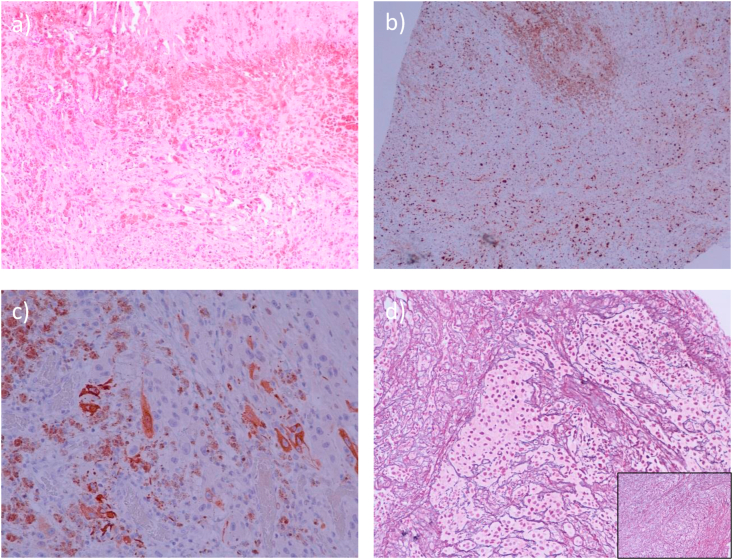
A) HE ×100 mag.: Heavily pigmented spindle cell neoplasm with epithelioid component (bottom) and necrosis (top). Psammoma bodies are absent. b) Ki67 × 40 mag.: Nuclear labelling index amounts to ~25% throughout [3 mitoses in one high power field (×40 objective), not illustrated]. c) MelanA x 200 mag.: Small clusters of immunopositive cells (on the right). In contrast, heavy intrinsic pigmentation on the left. Tumour cells contain oval, prominently nucleolated nuclei and well-defined basophilic cytoplasm with finely granular pigment consistent with neuromelanin. d) Reticulin x 100 mag. [b]: Prominent pericellular reticulin deposition overall but focal clusters of tumour cells (centre) lack pericellular wrapping. Inset: Prominent pericellular deposition in spindle cell areas.

CT at the time showed no evidence of peripheral metastases, whilst dermatology review revealed a 6mm asymmetrical ugly duckling naevus in the skin over the left knee [21]. There was no clinical evidence of Carney's complex. The skin lesion was then biopsied, which was diagnosed as a pT1a AJCC stage group 1a melanoma (Fig. 2). Wide local excision of the skin lesion was advised to ensure clear margins.

Re-exploration to achieve gross total resection of the extradural portion of the spinal tumour, as well as amputation of the C6 nerve root for local control was recommended. Pre-operatively, left vertebral artery occlusion was performed to reduce tumour vascularity and optimise the feasibility of achieving gross total resection. An anterior cervical approach was attempted but owing to significant tumour adherence, a subsequent revision posterior approach was required to accomplish gross total resection of the lesion and amputation of the nerve root.

### 2.6 Post-operative outcomes

After surgery, the patient experienced transient dysphagia due to post-surgical cervical haematoma, which responded well to conservative management with dexamethasone. Post-operative imaging confirmed excision of the majority of the residual enhancing lesion, with only a small residuum remaining. Adjuvant radiotherapy was commenced two months after surgery.

Three months following the completion of radiotherapy, the patient developed right-sided hip pain and subsequent imaging revealed a large metastasis in the right femoral neck that resulted in a pathological intracapsular fracture of the right femoral neck. A right hip hemiarthroplasty was thus carried out. In light of the bone metastasis, another staging CT was performed, revealing multiple lung metastases and possible liver metastasis. The patient was subsequently started on combination immunotherapy involving Nivolumab, an antibody against programmed death 1 (PD-1) receptor, Ipilimumab, an antibody against cytotoxic T-lymphocyte-associated antigen 4 (CTLA-4) as well as Denosumab, Receptor activator of nuclear factor kappa-Β (RANK) ligand-specific antibody [22,23].

Unfortunately, the patient's disease progressed despite treatment. She was readmitted to the hospital one month following the start of immunotherapy for bilateral pleural effusions, as well as pneumonia. Due to a rapid decline in her functional and performance status, palliative care and support was initiated. The patient passed away shortly after, within 15 months of diagnosis. The case report was written in retrospect and as such the patient's perspective was not feasible to be included.

### 2.7 Review of the literature and discussion

A systematic literature review was conducted across known databases, PubMed, EMBASE, Medline and reviewed the reported cases of extramedullary spinal MeS to date. A total of 65 cases were identified with the diagnosis of MeS (Table 1) across 46 published papers.

**Table 1:** Shows the number of MeS cases found in the literature. Level of lesion, age, gender, metastatic potential and T1-weighted and T2-weighted MRI imaging findings.

Level	Gender	Age	Reference	Metastasis	Region	MRI T1	MRI T2
**Cervical**							
C1	M	34	[6]	yes	Local and distal	–	–
C1	–	–	[19]	–	–	–	–
C1	M	54	[24]	no	N/A	hyperintense	hypointense
C2	F	30	[17]	yes	local	hyperintense	
C2-3	F	66	[25]^a^	–			
C2-3	F	35	[12]	yes	Local and distal	–	–
C4	M	36	[26]	yes	distal		
C4	–	–	[19]	–	–	–	–
C4-5	F	32	[27]	yes	distal	hyperintense	hypointense
C5-6	F	53	[28]	no	N/A	isointense	hyperintense
C6	F	26	[29]	no	N/A	–	–
C6-7	M	27	[30]	yes	local	–	–
C7	M	64	[10]	yes	Local and distal	hyperintense	hypointense
C8	F	49	[31]	–			
C8	F	49	[32]	no	N/A	–	–
**Thoracic**							
T1-T1	F	17	[18]	no	N/A	hyperintense	hypointense
T2	F	45	[33]^a^	–	–	–	–
T2	F	12	[31]^a^	–	–	–	–
T2-4	M	47	[34]	no	N/A	hyperintense	hypointense
T3	F	40	[35]	no	N/A		
T5	M	38	[36]	no	N/A	–	–
T6-7	M	40	[37]^a^	–	–	–	–
T6	M	34	[6]	yes	distal	–	–
T6-8	F	65	[38]	yes	local	hyperintense	–
T7	M	59	[39]	yes	local		
T7	M	25	[40]	yes	Local and distal	hyperintense	hypointense
T7	M	61	[41]^a^	–	–	–	–
T8-12	M	67	[42]	no	N/A	hyperintense	hypointense
T9	M	43	[12]	–	–	–	–
T9-10	M	53	[8]	no	N/A	hyperintense	hypointense
T10	F	58	[43]^a^	–			
T12-L1	F	17	[44]	no	N/A	–	–
T12-L2	–	–	[45]^a^	–	–	–	–
**Lumbar**							
L1	F	23	[41]^a^	–	–	–	–
L1	M	32	[12]	no	N/A	–	–
L1-2	M	43	[46]	no	N/A	hypointense	hyperintense
L1-2	M	22	[47]	no	N/A	hypointense	hyperintense
L1-2	F	75	[19]	–	–	–	–
L2	M	42	[19]	–	–	–	–
L2	M	37	[48]^a^	–	–	–	–
L1-5	F	35	[19]	–	–	–	–
L3	F	70	[49]^a^	–			
L3	F	46	[50]	yes	local	hyperintense	hyperintense
L3-5	F	35	[6]	yes	Local and distal	–	–
L4	M	59	[51]	yes	Local and distal	–	–
L4	F	40	[12]	yes	Distal and local	–	–
L4-5	M	60	[49]	–	–	hyperintense	hypointense
L5	M	27	[6]	yes	distal	–	–
L5-S1	M	28	[52]	yes	Local and distal	hyperintense	–
L5-S1	M	36	[53]	–	–	–	–
L5-S1	M	33	[38]	yes	Local and distal	–	–
L5-S1	F	36	[50]	yes	local	hyperintense	mixed
**Sacral**							
S1	F	26	[54]	no	N/A	–	–
S1	M	36	[51]^a^	–	–	–	–
S1	F	63	[18]^a^	–	–	–	–
S1	F	41	[6]	no	N/A	–	–
Level Unknown							
N/A	M	46	[55]	no	N/A	–	–
N/A	–	–	[8]	no	N/A	–	–
N/A	F	56	[56]	–	–	–	–
N/A	M	58	[56]	–	–	–	–
N/A	M	32	[57]^a^	–	–	–	–
N/A	M	35	**9**[19]	–	–	–	–
N/A	M	–	[58]	–	–	mixed	hypointense
N/A	M	–	[58]	–	–	mixed	hypointense
N/A	F	–	[58]	–	–	mixed	hypointense

a No access to full article or the article was not available in English.

**Fig. 3:**
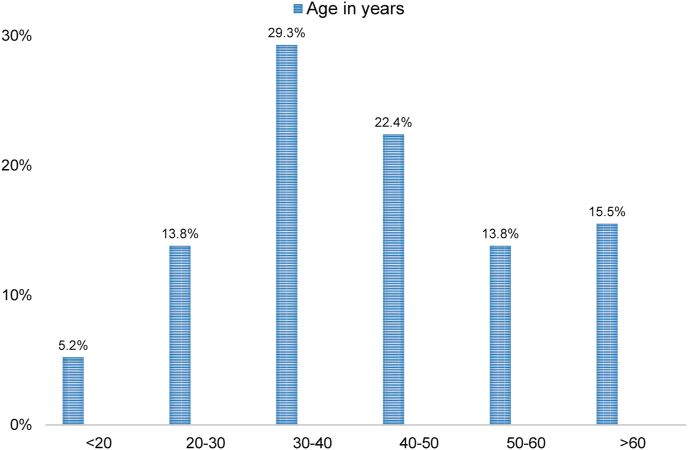
Percentage of patients versus age at diagnosis. Median age of presentation is 20–40 years of age.

In 61/65 (93.8%) cases, the gender was available, of which 33/61 (54.1%) were male, and 28/61 (45.9%) were female — patients with extramedullary MeS most commonly presented between 30 and 40 years of age (Fig. 3).

**Fig. 4:**
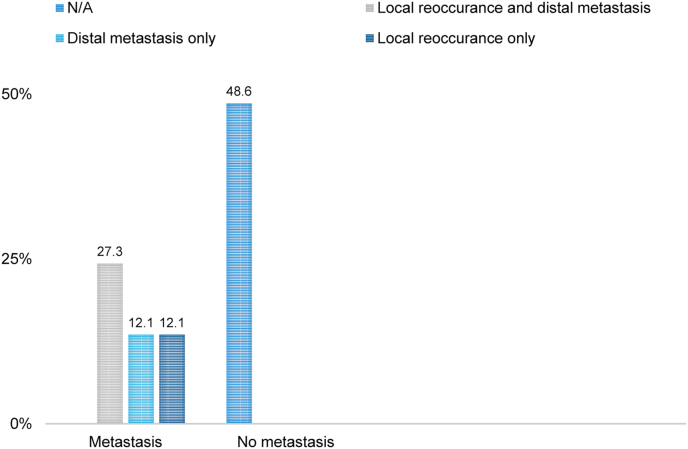
Percentage of patients versus metastasis and/or local reoccurrence. The majority of the cases show metastasis and/or local reoccurrence.

In 37/65 of the cases, information regarding metastasis was available. In 19/37 (51.4%) cases, evidence of metastasis or local recurrence was found, of which 9/37 (24.3%) had both distal metastasis and local recurrence, 5/37 (13.5%) had local recurrence in isolation, and 5/37 (13.5%) had distal metastasis in isolation (Fig. 4). In 18/37 (48.6%), no evidence of recurrence or metastases were noted. Notably, the follow-up time across studies was highly variable, ranging from a few months up to two years.

**Fig. 5:**
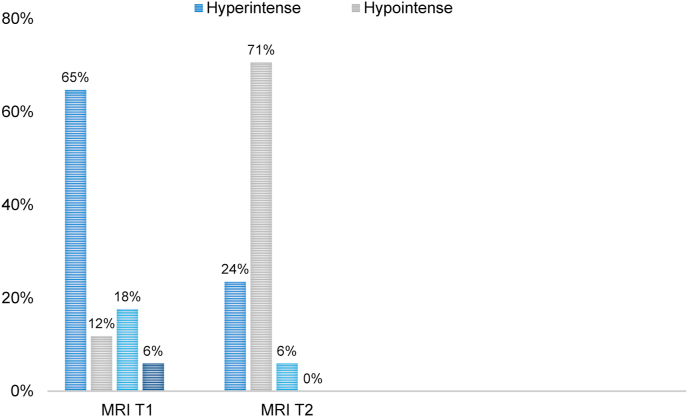
Percentage of patients with T1 & T2 MRI available versus signal intensity per sequence.

Regarding the radiological findings (Fig. 5), in 17/65 cases, both T1 and T2-weighted MRI sequences were available. The T1-weighted imaging was hyper-intense in the majority (11/17) with the rest demonstrating either mixed (3/17), hypo-intense (2/17) or iso-intense (1/17) signal changes. The T2-weighted imaging was predominantly hypo-intense (12/17) with a minority showing either hyper-intense (4/17) or mixed (1/17) signal intensities.

A number of case reports and a small number of case series share common thoughts regarding the histological classification, incidence and management of extramedullary MeS. According to the recent literature [34,50], there are two topics, which are still under discussion and subject to further evaluation regarding MeS. These are the T1-and T2-weighted MRI findings of MeS and the future prognosis regarding local recurrence and distant metastasis. According to our knowledge, there are no attempts to capture and summarise the radiological and prognostic evidence to date. Here, we endeavour to discuss our results in the context of the literature.

The literature review conducted, revealed similar age distribution to other studies, with 30–40 years of age being the most common age of presentation [6,7]. Moreover, the gender distribution revealed a 1.2:1 male to female ratio similar to other studies focusing on MeS [6,7,17].

It is widely accepted that MeS exhibit T1-weighted hyperintensity and T2-weighed hypointensity. On the contrary, schwannomas exhibit hypointensity on T1 and hyperintensity on T2. The review of 65 cases conducted, revealed that 64.7% of cases of MeS appear hyperintense, and 70.6% of cases appear hypointense in T1-and T2-weighted MRI sequences, respectively. Melanin exhibits paramagnetic effects, resulting in stable free radicals [[59], [60], [61]]. In essence, melanin protons have shorter T1 & T2 relaxation times. Therefore they quickly recover after an MRI pulse, and are able to appear brighter, leading to a hyperintense and hypointense T1 and T2 signals, respectively. The concentration of melanin and the density of the tumour are however not always consistent.

Notably, Liu et al. showed that 95% of primary spinal melanomas showed T1 and T2-weighted hyper and hypointensity, respectively [62]. Since higher melanin concentration was associated with higher T1-weighted signal intensities and lower signal intensities on T2-weighed images [61], tumours which have melanocytes as their cell origin might have a greater concentration of melanin compared to MeS. Thus, having even slower relaxation times and being less affected by the surrounding environment leads to higher specificity of T1 and T2 hyper- and hypointense signals, respectively.

Moreover, haemorrhage is common in CNS tumours [63]. Deoxy-haemoglobin and all iron-containing haeme groups exhibit paramagnetic effects. Timing of bleeding is also important: acute haemorrhage shows similar MRI findings to MeS (hyperintense T1 and hypointense T2), whereas subacute haematoma might show hyper-intense signal on T1 and hypo-intense T2-weighted images as well [64]. Therefore, melanin concentration and the extent and timing of haemorrhage are all of great significance to the MRI findings.

The identification of a coincidental cutaneous melanoma in our patient raised the question whether the skin lesion and the spinal tumour were causally related and whether the latter represented a metastatic malignant melanoma. However, the skin lesion was in an early, micro-invasive stage with very low metastatic potential. In addition, both radiological and histological features of the spinal tumour strongly favoured melanotic schwannoma over melanoma, and paraspinal location of metastatic melanoma is rare.

With regards to prognosis, an early literature review by Vallat-Decouvelaere [6] identified a metastatic disease in up to 26% of patients with MeS. More recently, Zhan et al. demonstrated that MeS could metastasize in 9.1% of the cases and recurred locally in 18.2% of the cases after resection [12]. Interestingly, Torres-Mora et al., in one of the largest studies on MeS to date, have found that MeS tumours are significantly more aggressive than previously thought, with local recurrence in 35% of the cases and evidence of metastasis reaching 44% [5]. The group concludes that MeS is a distinctive malignant tumour, rather than benign neoplasm with occasionally unpredictable behaviour, proposing its reclassification as “malignant melanotic schwannian tumour".

The comprehensive literature review conducted here, reveals that more than half of the cases (51.4%) are reported to have local or distal metastasis or both, confirming the suspicion that MeS is an aggressive neoplasm with high malignant potential. The aforementioned case report, written in accordance to the SCARE 2018 guidelines [ ], adds further value to the suspicion that MeS is an aggressive malignancy.

## 3 Conclusion

Melanotic schwannoma is a rare, aggressive and potentially malignant variant of schwannoma, one of the most commonly observed intradural extramedullary tumours in neurosurgical practice. A high degree of suspicion is required in the context of imaging findings demonstrating T1 hyperintensity and T2 hypointensity on MRI. Early screening with whole-body staging CT should be considered owing to the risk of distant metastases. The surgical strategy should be aimed at considering a gross total resection for optimal local control. This may necessitate intra-operative histological analysis by way of a frozen section to guide the extent of resection. Early follow-up and regular surveillance imaging are advisable. Further studies are required concerning adjuvant therapy in the treatment and whether this should be considered in all cases or only following a recurrence or metastatic disease.

## Funding

Not applicable.

## Availability of data and material

Table 1 includes all articles we used to conduct the literature review.

## Consent for publication

Written informed consent was obtained from the patient for publication of this case report and accompanying images. A copy of the written consent is available for review by the Editor-in-Chief of this journal on request.

## Provenance and peer review

Not commissioned, externally peer reviewed.

## Funding

No source of funding to declare.

## Ethical approval

Research studies involving patients require ethical approval. Please state whether approval has been given, name the relevant ethics committee and the state the reference number for their judgement. Patients consent was obtain prior to writing the case report.

## Author contribution

Please specify the contribution of each author to the paper, e.g. study concept or design, data collection, data analysis or interpretation, writing the paper, others, who have contributed in other ways should be listed as contributors.

GS, TN, SA, have all contributed to the study design, data collection, data analysis, drafting and structuring the case report.

WA and PU, have contributed in the data collection, analysis and writing the paper.

## Declaration of competing interest

No conflict of interest to declare.
